# Heterologous Expression of the Antiviral Lectin Griffithsin in Probiotic *Saccharomyces boulardii* and In Vitro Characterization of Its Properties

**DOI:** 10.3390/microorganisms12122414

**Published:** 2024-11-25

**Authors:** Jie Tang, Ran Li, Tingyu Jiang, Jiachen Lv, Yuwei Jiang, Xingjian Zhou, Hong Chen, Meiliang Li, Aimin Wu, Bing Yu, Timo M. Takala, Per E. J. Saris, Shuhong Li, Zhengfeng Fang

**Affiliations:** 1 Key Laboratory for Animal Disease-Resistance Nutrition of China Ministry of Education, Animal Nutrition Institute, Sichuan Agricultural University, Chengdu 611130, China; 2 Key Laboratory of Agricultural Product Processing and Nutrition Health (Co-Construction by Ministry of Agriculture and Rural Affairs of China and Sichuan Province), College of Food Science, Sichuan Agricultural University, Ya’an 625014, China; 3 Department of Microbiology, Faculty of Agriculture and Forestry, University of Helsinki, 00014 Helsinki, Finland

**Keywords:** *Saccharomyces boulardii*, griffithsin, next-generation probiotics, antiviral, heterologous expression

## Abstract

In this study, the probiotic yeast *Saccharomyces boulardii* was engineered to secrete the antiviral lectin griffithsin. Twelve genetic tools with the griffithsin gene were cloned into the vector pSF-TEF1-URA3 and introduced into *S. boulardii*. In the recombinant strains, a 16.9 kDa band was detected using SDS-PAGE and further recognized by griffithsin antibody with Western blotting. *S. boulardii* strains FM, FT, HC, and HE with a high yield of griffithsin were acquired for property characterization in vitro. The four recombinant strains displayed a similar growth pattern to that of the control strains, while their morphological characteristics had changed according to scanning electron microscopy. In simulated gastrointestinal digestive fluids, the survival rates of *S. boulardii* FM, FT, and HC were significantly decreased (86.32 ± 1.49% to 95.36 ± 1.94%) compared with those of the control strains, with survival rates between 95.88 ± 0.00% and 98.74 ± 1.97%. The hydrophobicity of *S. boulardii* FM, the strain with the highest griffithsin production, was significantly increased to 21.89 ± 1.07%, and it exhibited a reduced auto-aggregation rate (57.64 ± 2.61%). Finally, Vero cells infected with porcine epidemic diarrhea virus (PEDV) were used to evaluate the strains’ antiviral activity, and the rate at which *S. boulardii* FM inhibited PEDV reached 131.36 ± 1.06%, which was significantly higher than that of the control group.

## 1. Introduction

According to the FAO/WHO’s definition, probiotics are “live microorganisms, which when administered in adequate amounts, confer a health benefit on the host” [1]. The benefits of probiotic strains and their combinations have been evaluated extensively. The clinical significance of probiotics is mainly indicated by their roles in metabolic disorders (e.g., obesity and diabetes) [2,3], gastrointestinal disorders (e.g., antibiotic-associated diarrhea and constipation) [4,5], depression, anxiety, and mental disorders [6].

Even though probiotic strains have demonstrable functionality for certain diseases, they are currently still regulated as food or dietary supplements rather than drugs for disease control. However, the newly proposed concept of next-generation probiotics (NGPs) [7] may expand their usage to the field of disease control. One strategy for developing NGPs is to screen strains that have not traditionally been used as probiotics but that possess potential health benefits, especially for disease control. For example, the benefits of *Akkermansia muciniphila* [8], *Bacteroides xylanisolvens* DSM 23694 [9], *Bacteroides ovatus* D-6 [10], *Clostridium butyricum* MIYAIRI 588 [11,12], and *Faecalibacterium prausnitzii* [13,14] have previously been evaluated. Another strategy is using well-characterized probiotic strains as vehicles for the delivery of bioactive molecules like anti-inflammatory cytokines [15,16], antimicrobials [17], or even anticancer compounds [18]. In this way, these modified probiotic strains could exhibit dual effects of the molecules they secrete and probiotic benefits. Moreover, their well-characterized safety and tolerance to gastrointestinal pressure makes them ideal hosts for the in situ secretion of bioactive molecules into the intestine. NGPs also conform to the definition of LBPs, i.e., live biotherapeutic products, a concept officially approved by the FDA [19] and the European Directorate for the Quality of Medicines and Healthcare (EDQM) [20]. LBPs are defined as a biological product that “contains live organisms; is applicable to the prevention, treatment, or cure of a disease or condition of human beings; is not a vaccine” [19].

Given their dual recognition from academia and governments, screening or constructing live microorganisms that confer benefits for controlling diseases is becoming an emerging practice, and engineering well-characterized probiotic strains that can secrete disease-control-related molecules is an efficient method to this end [21]. A typical case is the bioengineered probiotic *Escherichia coli* Nissle 1917 (EcN 1917). For example, Gurbatri et al. [18] constructed an EcN 1917 variant that could colonize tumors for the detection and treatment of colorectal neoplasia, while Huang et al. [22] engineered EcN 1917 and evidenced its potential for development as an antiviral probiotic for controlling influenza.

As the only known probiotic yeast, *Saccharomyces boulardii* has been commercially available for 62 years [23], and it has been granted GRAS (generally recognized as safe) status by the FDA [24] and QPS (qualified presumption of safety) by the European Food Safety Authority (EFSA) [25]. In addition to its safety, *S. boulardii* is known for its superior resistance to proteolytic cleavage, acid, and heat, and its optimal growth temperature is 37 °C [26]. Intensive studies have revealed the efficacy of *S. boulardii* in regulating the symptoms of diarrhea [27,28,29,30,31,32,33,34,35], inflammatory bowel disease [36,37], irritable bowel syndrome [38], and *Helicobacter pylori* infections [39,40]. These features and benefits make *S. boulardii* a promising candidate for the delivery of bioactive molecules.

Meanwhile, griffithsin is a natural lectin from red algae that contains 121 amino acids. Its antiviral activity had been identified in previous research as strong (with an EC_50_ value in the picomolar range) and broad-spectrum [41], and it is capable of killing viruses including human immunodeficiency virus (HIV) [42], severe acute respiratory syndrome coronavirus (SARS-CoV) [43], Middle East respiratory syndrome coronavirus (MERS-CoV) [44], hepatitis C virus (HCV) [45,46], Japanese encephalitis virus (JEV) [47], and porcine epidemic diarrhea virus (PEDV) [48]. Considering the potential to apply griffithsin in disease control applications in humans or animals, in vitro and in vivo studies have been undertaken to confirm its lack of a toxic effect on the host [41].

In this study, we constructed antiviral NGPs in the form of griffithsin-secreting strains of *S. boulardii* and evaluated the properties of these bioengineered strains in vitro. The core idea was to combine the antiviral activity of griffithsin with the probiotic effects of *S. boulardii* and demonstrate their potential as carriers for the delivery of therapeutics in future applications.

## 2. Materials and Methods

### 2.1. Plasmids, Strains, and Media

The plasmids and strains used in this study are listed in Table 1. The intermediate host *E. coli* DH5α was grown in LB medium (1% tryptone, 0.5% yeast extract, and 1% sodium chloride) at 37 °C and supplemented with kanamycin (50 μg/mL) for the selection of the transformants. *S. boulardii* SAA940 was grown in YPD medium (1% yeast extract, 2% peptone, and 2% glucose) at 37 °C. The yeasts carrying vector or recombinant plasmids were grown in SD selection medium (6.7% yeast nitrogen base w/o amino acids, 2% glucose, and 1.29% Dropout Supplement Ura (Coolaber, Beijing, China)). No uracil was added to this medium in order to maintain the plasmids.

### 2.2. The Virus and Cell Model

PEDV and the host Vero cells were provided by Prof. Bing Yu (Animal Nutrition Institute, Sichuan Agricultural University). The Vero cells were grown in Dulbecco’s Modified Eagle Medium (DMEM) (Gibco, Carlsbad, CA, USA) supplemented with 10% FBS (Gibco). The cells were grown at 37 °C in a 5% CO_2_ incubator. The PEDV was grown and passaged in the Vero cells. Virus solution was mixed with trypsin (at a final concentration of 5 μg/mL), and the mixture was inoculated into Vero cells that had uniformly grown into a monolayer. After 90 min of PEDV adsorption, the virus mixture was discarded and replaced with serum-free DMEM for culturing to establish the PEDV-infected cell model.

### 2.3. The Griffithsin Gene and the Production Strategy

The DNA sequence of griffithsin was retrieved from GenBank under accession number AY744143.1 [42], comprising 366 bp nucleotides encoding 121 amino acids.

To optimize the production of griffithsin in *S. boulardii* probiotic yeast, codon usage optimization was first performed by GenScript (Nanjing, China). Second, a production strategy aiming to test different combinations of promoters and secretion signal sequences was designed in this study. Three native promoters (TEF1, TDH3, and PGK1) were identified from the genome of *S. boulardii* CNCM I-745 and selected to drive the expression of the griffithsin gene. Four secretion signal sequences (SED1, αMF, STA1, and CL) [49] were selected for the secretion of griffithsin, among which SED1 and αMF were native signal sequences identified from the genome of *S. boulardii* CNCM I-745. Detailed information on all the promoters and signal sequences is provided in the Supplementary Materials (Tables S1 and S2).

**Figure 1:**
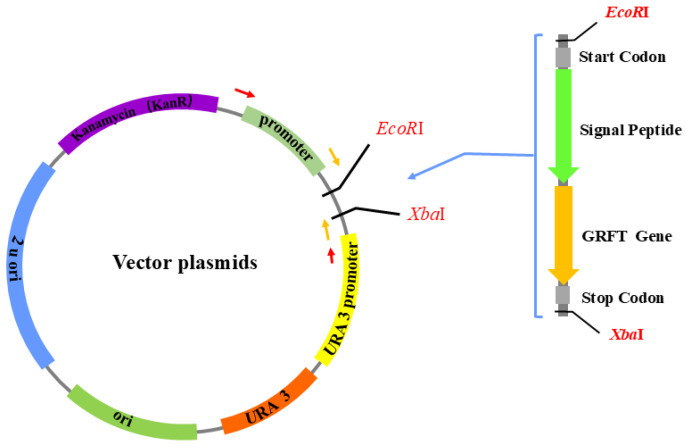
Cloning strategy for griffithsin expression plasmids. The gene expression cassette contained restriction enzyme sites (*Eco*RI and *Xba*I), a start codon (ATG) and a stop codon (TAA), signal sequences (SED1, αMF, STA1, and CL), and the griffithsin gene sequence (GRFT). The yellow arrows indicate the primer for colony PCR identification, and the red arrows indicate the primer for gene sequencing.

In order to generate plasmids with different promoters and signal sequences, first, the promoter in pSF-TEF1-URA3 (OG534) was replaced with three native promoters (TEF1, TDH3, and PGK1) from *S. boulardii* CNCM I-745 through homologous recombination. Subsequently, 4 gene expression cassettes containing the griffithsin gene and signal sequences were synthesized by GenScript and cloned into the 3 vectors, resulting in 12 different recombinant plasmids (Table 1). The cloning strategy is shown in Figure 1.

### 2.4. Construction of the Recombinant Yeast Strains

The recombinant probiotic yeast strains were constructed according to the procedures described in our previous studies [17,50]. Briefly, an empty vector or plasmids with different promoters and signal sequences were introduced into the intermediate host *E. coli* DH5α using electroporation. Then, the plasmids were isolated and electroporated into *S. boulardii* SAA940. Transformants were selected on SD agar, and the colonies were verified using PCR with plasmid-specific primers (F, 5′-AACTGCTGATCGAGTGTAGCCAG-3′; R, 5′-ATCAGTCAGTCAGTGCAGGAGGA-3′) to amplify fragments from 1136 bp to 1728 bp depending on the size of the promoters and the gene expression cassettes (the primer binding sites are indicated by yellow arrows in Figure 1). After each transformation, sequencing was carried out to ensure no mutations were generated in the DNA sequences of the promoters or the gene expression cassettes (the primer binding sites are indicated by red arrows in Figure 1). The forward primer for sequencing was 5′-AACTGCTGATCGAGTGTAGCCAG-3′, and the reverse primer was 5′-ATCAGTCAGTCAGTGCAGGAGGA-3′.

### 2.5. Secretion of Griffithsin and Western Blot Analysis

After PCR and sequencing identification, twelve recombinant yeast strains carrying the correct plasmids were selected as the hosts for griffithsin secretion. All of the recombinant yeast strains were inoculated into SD medium, and the wild-type strain *S. boulardii* SAA940 was inoculated into YPD medium. All the strains were incubated at 37 °C and shaken for 72 h. A total of 50 milliliters of supernatant was collected using centrifugation (5000× *g*, 5 min) and filtered with a 0.22 μm filter (Merck Millipore, Darmstadt, Germany). Afterwards, the cell-free supernatant was concentrated using an Amicon Ultra-15 Centrifugal Filter (3000 NMWL, Merck Millipore, Darmstadt, Germany).

The collected samples were subjected to SDS-PAGE and Western blot analysis to preliminarily verify the secretion of griffithsin. A BeyoGel™ Plus PAGE precast gel (Tris-Gly System, 15% resolving gel) (Beyotime, Shanghai, China) was applied for the SDS-PAGE analysis. Western blotting was subsequently performed following the basic procedures previously described by us [51], except that the first antibody was an anti-griffithsin antibody (1:1000 dilution, CUSABIO, Wuhan, China), and the incubation and washing steps were performed in an automatic blotting system (BLT WB-600 Auto, Biolight, Guangzhou, China). Final visualization was performed using an enhanced chemiluminescence kit (BeyoECL Plus, Beyotime, Shanghai, China) and the ChemiDoc Imaging System (Bio-Rad, Hercules, CA, USA).

### 2.6. Confirmation of the High-Production Strains

To identify strains that secreted high levels of griffithsin, first, the total protein concentration of all of the aforementioned samples was measured using the BCA method [52]. Then, the samples were adjusted to the same protein concentration and subjected to SDS-PAGE at the same volume. Given the same amount of total protein was used, the griffithsin yields between different strains could be compared according to Quantity One (version 4.6.2, Bio-Rad, Hercules, CA, USA) analysis [51].

### 2.7. Growth Profiles

After the construction and verification of the recombinant yeasts, strains with high griffithsin yields were selected for the further characterization of their properties. Their growth profiles were first investigated by generating time–OD_600_ curves. Briefly, a single colony was picked for each strain, inoculated into the corresponding medium, and cultured overnight. The next day, the fresh overnight cultures were adjusted to the same OD_600_ value and then transferred into pre-warmed medium with 2% inoculum for continuous incubation. Samples were collected every 2 h, and their OD_600_ values were monitored using a spectrophotometer (759S, Lingguang, Shanghai, China) to generate the growth curves.

### 2.8. Morphology

Before observing their morphology, the yeast strains were cultured to the exponential phase. Their overall morphology, such as their size, shape, and density, was observed after methylene blue staining using an optical microscope. To observe possible changes in the micro-structure of the yeast strains further, a scanning electron microscope (SEM) was used to capture the details of the yeast cell surfaces.

### 2.9. Tolerance to Gastrointestinal Pressure

An in vitro simulation was used to study the tolerance of each strain to gastrointestinal pressure. Simulated digestive fluids, including gastric fluid, small intestine fluid, and large intestine fluid, were prepared as described by Regmi et al. [53]. The yeast strains were pre-cultured for 24 h and added into the digestive fluids mentioned above at a ratio of 1:10. Then, the mixture was incubated at 37 °C and shaken. Samples were collected from the gastric fluid at 1 h and 2 h; from the small intestine fluid at 2 h and 4 h; and from the large intestine fluid at 2 h, 4 h, 6 h, and 8 h [54]. After simulating digestion, the samples were serially diluted to determine the colony forming units (CFU). The survival rate of each yeast strain was calculated as follows: survival rate% = (post-digestion CFU/initial CFU) × 100%.

### 2.10. Hydrophobicity and Auto-Aggregation

The hydrophobicity of the yeasts’ surface was evaluated by employing a test of microbial adhesion to hydrocarbons [55]. The overnight yeast cultures were centrifuged at 5000× *g* for 10 min, after which the supernatant was removed. The pelleted cells were rinsed with sterile phosphate-buffered saline (PBS) and re-suspended to a concentration of 10^8^ CFU/mL. This adjustment resulted in an optical density (600 nm) of the yeast suspension that was standardized to 0.5 ± 0.05, referred to as A0. An equal volume of xylene was then added to the cell suspension, and they were mixed thoroughly using a vortex. The mixture was allowed to stand for 10 min at room temperature, followed by 30 min of incubation at 37 °C to facilitate phase separation. Subsequently, the aqueous phase was gently aspirated, and its optical density at 600 nm was recorded (A1). The percentage of cell surface hydrophobicity (H%) was calculated using the formula H% = [1 − (A1/A0)] × 100%.

Their auto-aggregation rates were also evaluated. The yeast cells were subjected to centrifugation at 5000× *g* for 10 min. Then, the supernatant was discarded, and cell pellets was harvested. The cells were washed twice with PBS and re-suspended into the same volume of PBS, after which the OD_600_ value was measured (A0). Subsequently, a 2 mL aliquot of the cell suspension was vortexed for 10 s and then allowed to stand for 2 h at 37 °C. After this interval, a 1 mL portion of the supernatant was taken, and its absorbance (A2) at 600 nm was quantified. The percentage of auto-aggregation was calculated using the formula Auto-aggregation (%) = [1 − (A2/A0)] × 100% [55].

### 2.11. The Antiviral Activity Assay

The strain *S. boulardii* FM was chosen as the candidate for assessing the antiviral capabilities of the recombinant yeasts. PEDV served as the model virus. The Vero cells were seeded into 96-well plates, and PEDV was introduced into them, as detailed in Section 2.2, to establish a PEDV-infected cell model (the virus control). Two preparations of *S. boulardii* FM were utilized for co-incubation with PEDV within the yeast group, (i) cell broth containing both yeast cells and their metabolites and (ii) yeast cells alone, with the supernatant removed after centrifugation. The mixture of PEDV and *S. boulardii* FM was incubated for 2 h at 37 °C, after which it was transferred into pre-cultured Vero cells for an additional 90 min of incubation. A batch of control cells, i.e., Vero cells without PEDV or yeast treatment, was also cultured. The CCK-8 assay was employed to determine the OD_450_ value of the viable Vero cells post-treatment. The antiviral activity was quantified by the PEDV inhibition rate (%), calculated as follows: (OD_450_ value of yeast group-OD_450_ value of virus control)/(OD_450_ value of cell control-OD_450_ value of virus control). The empty vector strain *S. boulardii* OG534 was used in the same way to assess the effects of native *S. boulardii* yeast on PEDV.

### 2.12. Data Analyses

The data collected were analyzed using SPSS Statistics 22.0 (IBM, Armonk, NY, USA), and one-way analysis of variance (ANOVA) was performed for statistical assessment. For the post hoc test, Duncan’s method was used for multiple comparisons between multiple groups; when only two groups were compared, the independent sample *t*-test was used. A difference was considered significant when the *p* value was less than 0.05.

## 3. Results

### 3.1. Cloning and Verification of the Gene Expression Cassettes

As listed in Tables S1 and S2, we first identified two indigenous signal sequences from the genome of *S. boulardii* CNCM I-745: SED1 located on chromosome IV (GenBank accession number CM003540.1, 630940–630996 bp) and αMF located on chromosome XVI (GenBank accession number CM003552.1, 33929–34195 bp). Additionally, we utilized two exogenous signal sequences: STA1 from *Saccharomyces diastaticus* [56] and a chicken lysozyme signal sequence (CL) [49,57]. Concurrently, we identified three native promoters: PGK1 on chromosome III (GenBank accession number CM003539.1, 135935–136891 bp); TDH3 on chromosome VII (GenBank accession number CM003543.1, 198933–199576 bp); and TEF1 on chromosome XVI (GenBank accession number CM003552.1, 539901–540478 bp).

**Figure 2:**
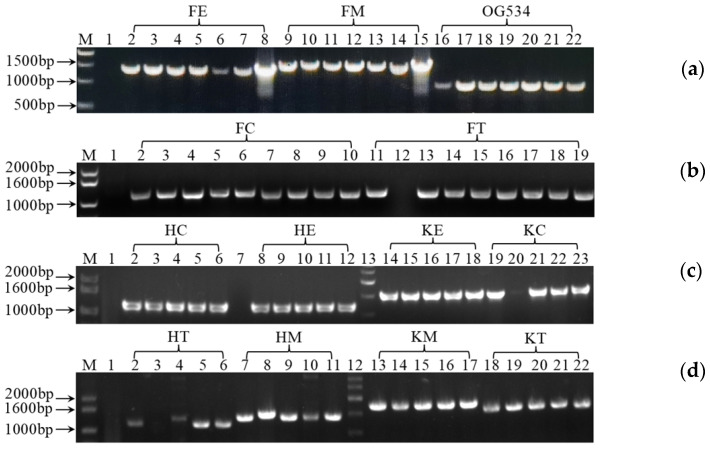
Verification of recombinant strains by analyzing DNA fragments after PCR amplification. M: DNA molecular weight marker, 500 bp DNA Ladder and 1 kb plus DNA Ladder (TianGen, Beijing, China). Lane 1 in gel pictures (**a**–**d**): blank control for PCR. (**a**) Lanes 2–8: PCR results using *S. boulardii* FE as the template (expected size: 1424 bp); lanes 9–15: *S. boulardii* FM (1634 bp); lanes 16–22: *S. boulardii* OG534 (765 bp); vector control. (**b**) Lanes 2–10: *S. boulardii* FC (1136 bp); lanes 11–19: *S. boulardii* FT (1172 bp). (**c**) Lanes 2–6: *S. boulardii* HC (1202 bp); lanes 8–12: *S. boulardii* HE (1205 bp); lanes 14–18: *S. boulardii* KE (1518 bp); lanes 19–23: *S. boulardii* KC (1515 bp). (**d**) Lanes 2–6: *S. boulardii* HT (1238 bp); lanes 7-11: *S. boulardii* HM (1415 bp); lanes 13-17: *S. boulardii* KM (1728 bp); lanes 18–22: *S. boulardii* KT (1551 bp).

Subsequently, we examined the efficiency of the selected genetic tools in controlling the gene expression and secretion of griffithsin. To this end, 12 recombinant plasmids were constructed, as listed in Table 1, that contained either endogenous or exogenous genetic tools. After enrichment in the intermediate host *E. coli* DH5α, the isolated plasmids were transferred into the probiotic yeast, and the transformants were selected on SD agar. As shown in Figure 2, the selected yeast transformants displayed bands of different sizes on the agarose gel after PCR amplification. Compared with the vector control *S. boulardii* OG534, which presented 765 bp from the plain vector pSF-TEF1-URA3 (OG534), the transformants that contained the recombinant plasmids produced larger bands depending on the size of the inserted gene expression cassettes. These results verified the insertion of the gene expression cassettes and the presence of the recombinant plasmids in the yeast transformants. Based on these PCR results, plasmids of the right size were isolated and sent for sequencing. Yeast strains carrying recombinant plasmids without DNA mutations were subjected to our subsequent protein secretion steps.

### 3.2. Identification of the Secreted Griffithsin

**Figure 3:**
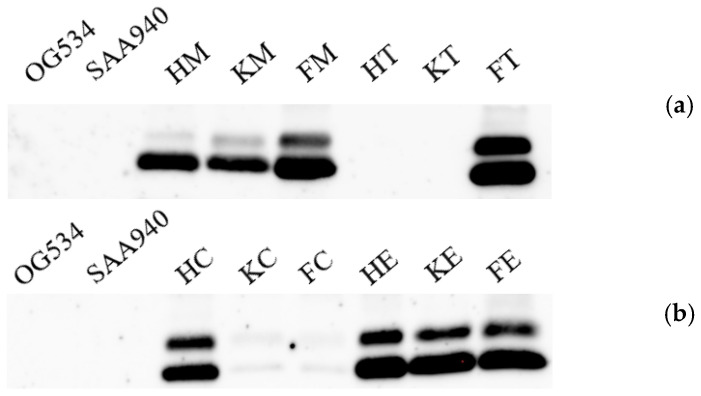
Western blotting of secreted griffithsin in cell-free supernatants of recombinant yeast cultures. OG534 and SAA940 in (**a**,**b**): supernatant from *S. boulardii* OG534 and *S. boulardii* SAA940. (**a**) HM: *S. boulardii* HM; KM: *S. boulardii* KM; FM: *S. boulardii* FM; HT: *S. boulardii* HT; KT: *S. boulardii* KT; FT: *S. boulardii* FT. (**b**) HC: *S. boulardii* HC; KC: *S. boulardii* KC; FC: *S. boulardii* FC; HE: *S. boulardii* HE; KE: *S. boulardii* KE; FE: *S. boulardii* FE.

Upon successfully acquiring the transformants carrying the correct plasmids, we proceeded to cultivate 12 recombinant yeast strains for the production of griffithsin. The supernatant was collected from each strain, and Western blotting was employed to identify the presence of secreted griffithsin within the total secreted protein profile of the host, as illustrated in Figure 3. The absence of specific bands in the control strains *S. boulardii* OG534 (Figure 3a,b) and *S. boulardii* SAA940 (Figure 3a,b) confirmed that neither griffithsin nor its derivatives are secreted by the native host.

Notably, two bands were observed from the recombinant strains of *S. boulardii* FM, FT, HC, HE, KE, and FE. In addition, a faint band was identified above the most prominent band for *S. boulardii* strains HM and KM. These results revealed the secretion of griffithsin by these yeast strains due to the introduction of the recombinant plasmids. Conversely, in the *S. boulardii* strains HT, KT, KC, and FC, the absence of proper bands was suggestive of either failed secretion or a very low level of griffithsin production.

### 3.3. Selection of the Yeast Strains with High Griffithsin Production

**Figure 4:**
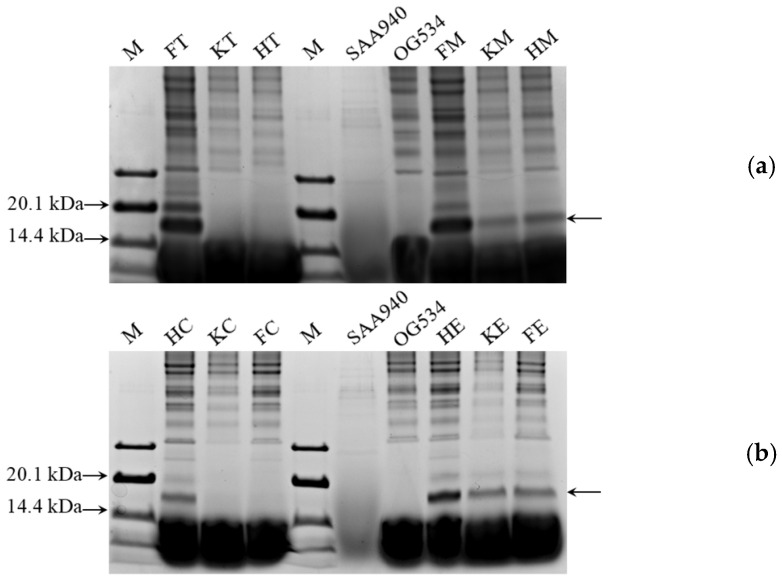
SDS-PAGE analysis of the cell-free supernatants from different recombinant yeast strains. M: Pre-stained ultra-low-molecular-weight protein marker (3.3 kDa–31.0 kDa, Solarbio Science & Technology, Beijing, China). OG534 and SAA940 in (**a**,**b**): total proteins of *S. boulardii* OG534 and *S. boulardii* SAA940. (**a**) FT: *S. boulardii* FT; KT: *S. boulardii* KT; HT: *S. boulardii* HT; FM: *S. boulardii* FM; KM: *S. boulardii* KM; HM: *S. boulardii* HM. (**b**) HC: *S. boulardii* HC; KC: *S. boulardii* KC; FC: *S. boulardii* FC; HE: *S. boulardii* HE; KE: *S. boulardii* KE; FE: *S. boulardii* FE. A band corresponding to griffithsin is marked by a black arrow.

In addition to quantifying the relative levels of griffithsin produced, our SDS-PAGE results also confirmed that griffithsin had been secreted, as indicated by the presence of a distinct band in Figure 4a,b (marked by black arrows). An additional band of around 16.9 kDa, as predicted using Quantity One, was detected in eight of the yeast strains (*S. boulardii* FT, FM, KM, HM, HC, HE, KE, and FE) but was absent in the control *S. boulardii* strains SAA940 and OG534. In the *S. boulardii* strains KT, HT, KC, and FC, no band corresponding to griffithsin was identified, in concordance with the findings of the Western blotting analysis detailed in Section 3.2.

**Figure 5:**
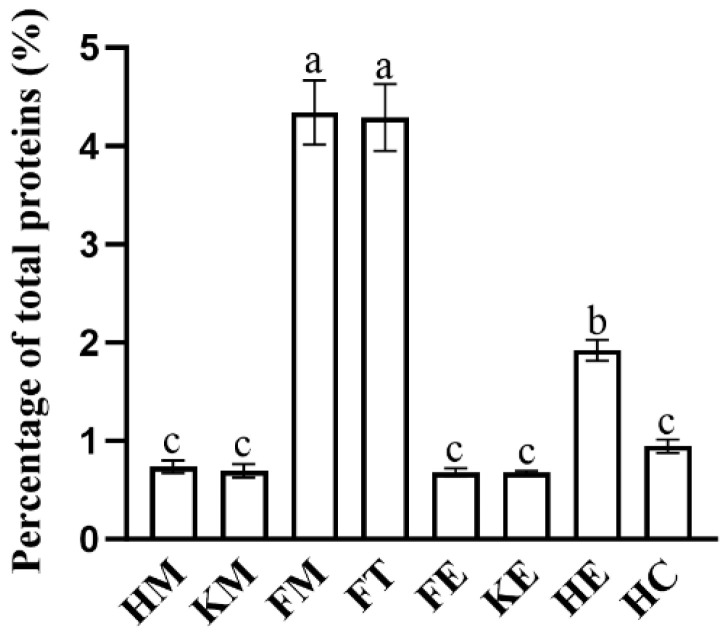
The relative percentage of griffithsin in the total proteins secreted by the yeast strains. Based on band intensities in SDS-PAGE gel, grayscale analysis was conducted to determine the relative yield of griffithsin in each recombinant strain. Data are presented as mean values with standard deviation. Duncan’s method was used for multiple comparisons of data between different groups; different letters indicate significant differences between different groups (*p* < 0.05).

Another reason for using SDS-PAGE was to select yeast strains that produced high levels of griffithsin. Prior to loading the samples onto the SDS-PAGE gel, the protein concentrations of all the samples were standardized to an equivalent level, and they were loaded at the same volume. By using a protein marker with known band concentrations as a benchmark, the relative percentage of griffithsin in the total proteins secreted by the yeast strains was calculated through grayscale analysis. As depicted in Figure 5, *S. boulardii* FM and FT emerged as the top two producers of griffithsin, followed by the strains HE and HC. Consequently, these four strains were prioritized for further characterization of their properties.

### 3.4. Characterization of the Properties of Selected Yeast Strains

#### 3.4.1. Growth Characteristics of the *S. boulardii* Strains

**Figure 6:**
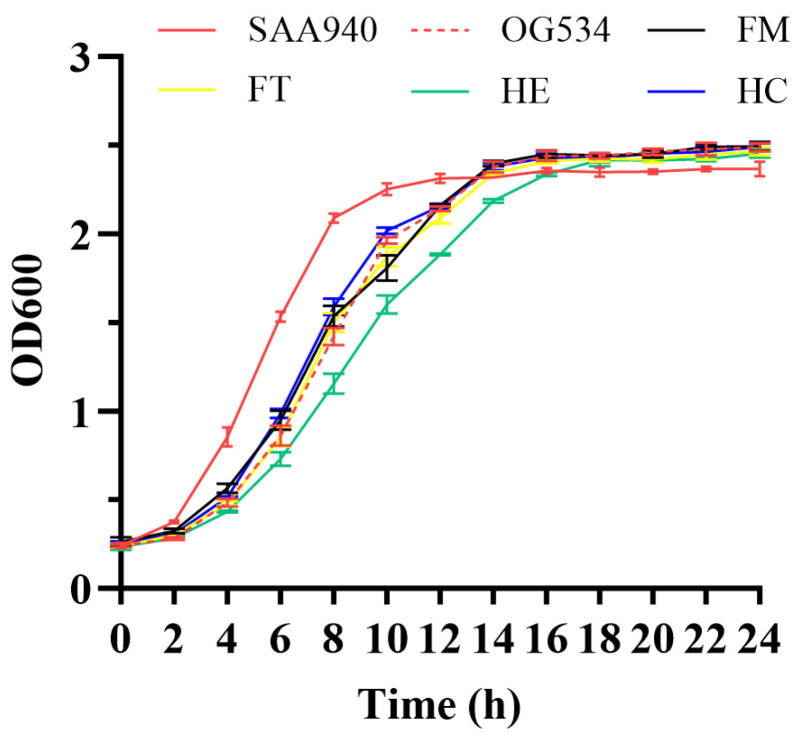
Growth curves of selected yeast strains. The strains were grown for 24 h, and the optical density (OD_600_) of the cultures was determined by a spectrophotometer every two hours after inoculation. Data are presented as mean values with standard deviation.

To determine potential alterations in the characteristics of the yeasts upon the introduction of exogenous plasmids and the secretion of griffithsin, first, their growth profiles were investigated. In general, all the strains tested achieved a comparable optical density at 600 nm of approximately 2.5 after 24 h of cultivation (Figure 6). However, the strains harboring exogenous plasmids, either carrying the plain vector (*S. boulardii* OG534) or recombinant plasmids (*S. boulardii* FM, FT, HE, and HC), exhibited a lag in the exponential growth phase when compared to the *S. boulardii* SAA940 strain. Additionally, it appeared that the secretion of griffithsin did not affect the growth of the recombinant strains, as the growth patterns observed in strains FM, FT, and HE were similar to those of the vector control strain *S. boulardii* OG534.

#### 3.4.2. Morphological Characteristics of the *S. boulardii* Strains

**Figure 7:**
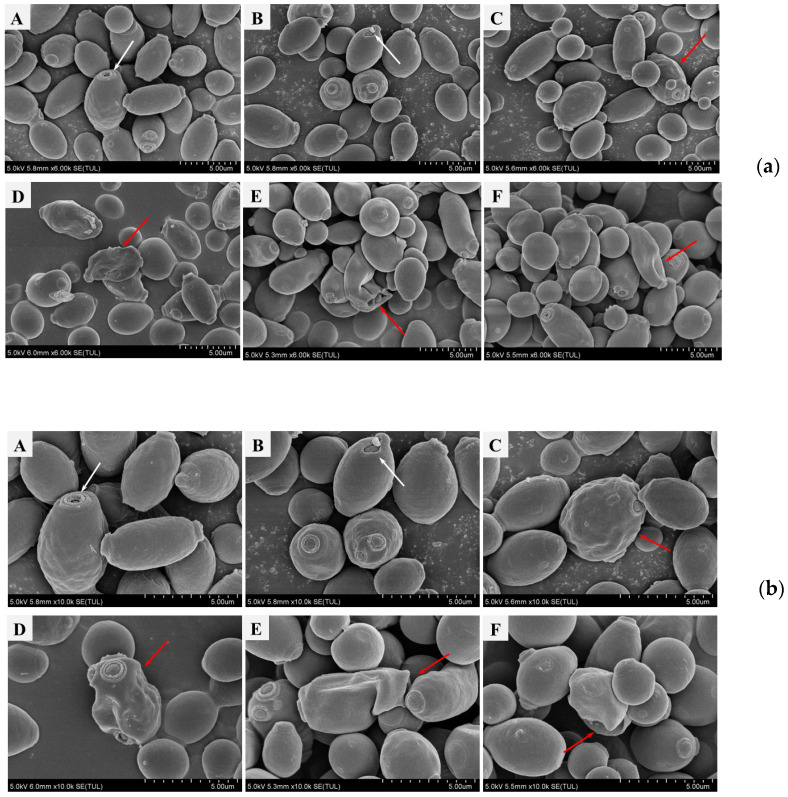
Scanning electron microscopy of selected yeast strains. Strains were cultured to the same growth phase with the same OD_600_ value, and pre-treated for SEM. Pictures were captured with magnification of 6000× (**a**) and 10,000× (**b**). A: *S. boulardii* SAA940; B: *S. boulardii* OG544; C: *S. boulardii* FM; D: *S. boulardii* FT; E: *S. boulardii* HE; F: *S. boulardii* HC. White arrows indicate indentations resulting from budding reproduction in the control strains, while red arrows indicate irregular morphological features in the recombinant strains.

Alterations in the morphology of the selected yeast strains were examined under an SEM. At the same growth stage, the control strains *S. boulardii* SAA940 (Figure 7a,b, picture A) and OG534 (Figure 7a,b, picture B) exhibited the ovoid or spherical cell shape characteristic of *Saccharomyces* species; no significant changes in the cell surfaces were noted, with the presence of minor indentations resulting from budding reproduction constituting the only discernible feature, as indicated by the white arrows in Figure 7. In contrast, the four recombinant strains (Figure 7a,b, picture C–F) had irregular morphological features, such as collapsed cells, a roughened surface, and an irregular outline. These morphological anomalies are highlighted with red arrows, and these distinctions became more pronounced under 10,000× magnification (Figure 7b).

#### 3.4.3. Simulation of Gastrointestinal Stress Tolerance In Vitro

To assess the ability of the selected yeast strains to withstand gastrointestinal conditions, artificial digestive fluids that mimicked the pH and enzymatic activity of natural digestive fluids were formulated. The survival rates of the tested strains following their incubation with these fluids are compiled in Table 2. The two control strains exhibited comparable survival patterns across the three types of digestive fluids, with these rates consistently hovering between 95.88 ± 0.00% and 98.74 ± 1.97%. This high level of survival underscores the excellent resilience of the native probiotic strain *S. boulardii* to stresses in the gastrointestinal tract. However, the production of griffithsin notably impacted the survival of certain strains of *S. boulardii*, specifically FM, FT, and HC. These strains demonstrated a significantly lower survival rate, ranging from approximately 86.32 ± 1.49% to 95.36 ± 1.94%, compared to that of the control strains (*p* < 0.05).

#### 3.4.4. Evaluation of Their Hydrophobicity and Auto-Aggregation In Vitro

**Figure 8:**
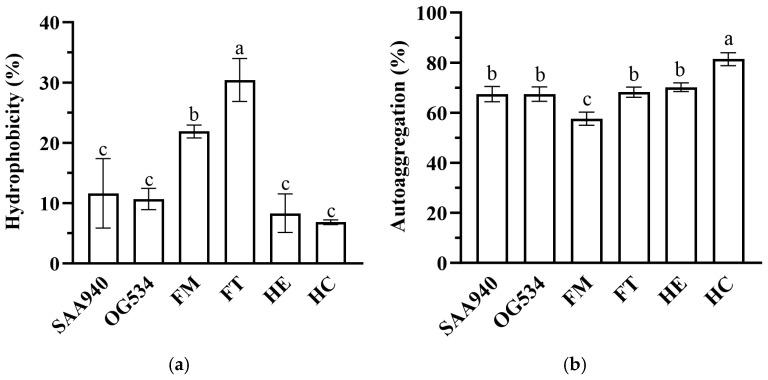
Hydrophobicity (**a**) and auto-aggregation (**b**) of the selected yeast strains. Surface hydrophobicity of the yeast strains was examined by determining the cells’ adhesion to hydrocarbon xylene. Auto-aggregation was evaluated by letting the yeast cells in a homogenous suspension settle down for 2 h, and measuring the OD_600_ from the supernatant. Data are presented as mean values with standard deviation. Duncan’s method was used for multiple comparisons of data between different groups; different letters indicate significant differences between different groups (*p* < 0.05).

The hydrophobicity and auto-aggregation of the selected strains were evaluated. As shown in Figure 8a, the control strains *S. boulardii* SAA940 and OG534 exhibited hydrophobicity values of 11.65 ± 5.76% and 10.69 ± 1.77%, respectively. Similarly, the *S. boulardii* strains HE and HC demonstrated relatively low levels of hydrophobicity of 8.33 ± 3.21% and 6.85 ± 0.38%. Notably, the two strains that produced higher levels of griffithsin exhibited hydrophobicity values that were more than double those in the other strains (*p* < 0.05), reaching 30.45 ± 3.56% for *S. boulardii* FT and 21.89 ± 1.07% for *S. boulardii* FM.

As illustrated in Figure 8b, the auto-aggregation results indicated that four of the tested strains showed comparable levels of auto-aggregation, with these values ranging from 67.08 ± 4.26% to 70.19 ± 1.70%. However, variations were observed in the *S. boulardii* strain FM, in which the lower value of 57.64 ± 2.61% (*p* < 0.05) was exhibited, and the HC strain, which showed a higher auto-aggregation rate of 81.38 ± 2.58% (*p* < 0.05).

#### 3.4.5. Anti-PEDV Activity In Vitro

**Figure 9:**
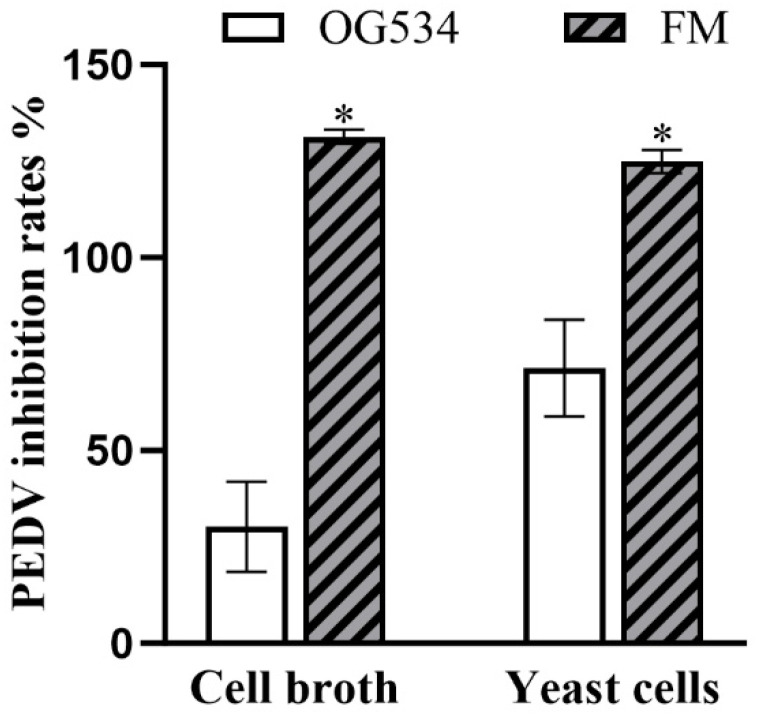
Rates of PEDV inhibition. Cell broth and yeast cells of *S. boulardii* FM and OG534 were pre-incubated with PEDV and then added to Vero cells for virus infection. Vero cells with or without infection were used as virus control and cell control, respectively. OD_450_ values of viable cells were measured using CCK-8 assay. PEDV inhibition rates% = (OD_450_ value of yeast group-OD_450_ value of virus control)/(OD_450_ value of cell control-OD_450_ value of virus control). Data are presented as mean values with standard deviation. The independent sample *t*-test was used when only two groups were compared; “*” indicates significant differences between two groups (*p* < 0.05).

An in vitro model of PEDV-infected Vero cells was applied in this study to evaluate the antiviral activity of the yeasts. *S. boulardii* yeast was first diluted to its maximum non-toxic concentration (unpublished data) to exclude any potential adverse effects on the Vero cells. After co-incubation, the tested *S. boulardii* strain FM displayed rates of inhibition of PEDV of 124.94 ± 1.71% (in the yeast cell group) and 131.36 ± 1.06% (in the cell broth group) (Figure 9), values which were significantly higher than those from the native yeast control (30.22 ± 6.76% and 71.35 ± 7.27%, *p* < 0.05).

## 4. Discussion

In addition to the safety and efficacy of *S. boulardii* referred to in the Introduction, as a yeast, it does not participate in horizontal gene transfer with intestinal bacteria. Together, these attributes make it an ideal microorganism to engineer into an NGP and for further development as an LBP as a biotherapeutic of the future.

Most of the existing studies on *S. boulardii* have focused on evaluating its probiotic effects and possible mechanisms, especially for the strain *S. boulardii* CNCM I-745 [27,35,36,40,58]. Mining out genetic manipulation tools is crucial for engineering *S. boulardii* into NGPs. Therefore, first, several genetic manipulation tools native to *S. boulardii* CNCM I-745 were located and identified in this study (Tables S1 and S2). Then, by cloning selected tools in different combinations, we found the most efficient combinations for regulating the secretion of griffithsin (Figure 5). Using these native tools reduces the chance of introducing foreign DNA fragments when genetically engineering *S. boulardii.* Because auxotrophic strains and appropriate genetic tools were lacking in the past, antibiotic selection was often applied in early attempts to genetically modify *S. boulardii* [17,59,60,61,62]. However, to ensure the safety of prospective applications of *S. boulardii* as an LBP, removing antibiotics from the engineering process is imperative. In this study, we employed the *URA3* auxotrophic strain of *S. boulardii* CNCM I-745 from Bagherpour et al. [63], specifically *S. boulardii* SAA940, as the host. The same host strain, transformation method, and screening strategy were also applied in a previous study by us [50]. Likewise, Liu et al. [57] and Bagherpour et al. [63] used the *URA3* auxotrophic strain of *S. boulardii* as their host and obtained comparable results in terms of heterologous expression. Our results have once again confirmed the effectiveness and feasibility of this host strain, which can be transformed and screened easily, as illustrated in Figure 2.

Two main ways to develop *S. boulardii* into an NGP have previously been applied: according to its secretion of functional substances [17,49,50,57,62,64,65,66] or its modification into an antigen delivery vehicle [60,63,67]. In our case, the idea was to modify *S. boulardii* so that it secreted antimicrobial substances. The successful secretion of griffithsin was confirmed by us through Western blotting (Figure 3) and SDS-PAGE (Figure 4) analyses. Interestingly, two bands were recognized by the griffithsin antibody in the Western blotting analysis, an observation previously revealed in another study by Vamvaka et al. [68], in which the griffithsin produced in rice endosperm also generated two bands in immunoblotting. This phenomenon could be attributed to post-translational modifications by a eukaryotic host, a process that is not typically observed in most prokaryotic expression systems [48,69]. Another explanation might be incomplete removal of the signal sequences [70]. Most importantly, the pattern of griffithsin secretion observed in this study did not affect its antiviral activity, as evidenced in Figure 9.

Properties pertaining to the production, consumption, and intestinal interaction of probiotics are crucial to fully harnessing their benefits. Consequently, we examined the characteristics of the modified *S. boulardii* strains to assess any potential alterations induced in them by genetic engineering. The growth curves (Figure 6) suggested that the auxotrophic selection employed in this study did not disrupt the growth of the recombinant yeast strains. Morphology, hydrophobicity, and auto-aggregation were studied to reflect the properties of the cell surfaces of the yeast strains. The secretion of griffithsin indeed changed the morphology of the recombinant strains (Figure 7), possibly due to the high yield of heterologous proteins, the growth conditions of the host, or the proteins accumulated on the cell surfaces. The hydrophobicity results (Figure 8a) support the protein accumulation theory, as the significantly enhanced hydrophobicity observed for *S. boulardii* FM/FT (*p* < 0.05) may have been caused by griffithsin adhering to its cell surfaces. While the hydrophobicity of griffithsin itself is unknown, its stability in organic solvents suggests that it is robustly hydrophobic [41]. In general, the native strain of *S. boulardii* demonstrated a good level of self-aggregation, as observed using methylene blue staining (Supplementary Materials Figure S1), in which the yeast cells tended to cluster together. This characteristic is also quantitatively reflected in Figure 8b, and it could be seen that different levels of griffithsin expression had varying impacts on the ability of the recombinant strains to self-aggregate.

Its exceptional tolerance to gastrointestinal stress is the crucial property that positions *S. boulardii* as the only probiotic type of yeast. After their genetic modification, the recombinant yeast strains still showed remarkable survival rates in the different digestive fluids (Table 2). This was consistent with our findings from previous research [17]. The two strains with the highest expression levels, *S. boulardii* FM and FT, exhibited lower survival rates compared to those of the other strains, indicating that although griffithsin expression did not affect the ability of the recombinant strains to grow normally, it still represented a burden when they underwent certain harsher conditions.

Finally, we utilized PEDV as a model virus to assess the antiviral activity of the recombinant yeast *S. boulardii* FM. Despite the fact that the griffithsin expressed in this yeast was not purified or concentrated but rather used directly with live yeast cells and their metabolites in the antiviral experiments, we still achieved promising results (Figure 9). This indicates the feasibility of employing probiotics as hosts for the secretion and delivery of antiviral agents. Future studies could focus on evaluating the antiviral efficacy of recombinant yeast using animal models or employing more stable genetic modification methods, such as CRISPR-Cas9, to construct NGPs with more stable expression.

## 5. Conclusions

This study systematically investigated the enhancement of the probiotic *S. boulardii* into an NGP with antiviral capabilities. Our findings confirmed that the transformation and screening methodologies established by us in prior research were both reliable and efficacious for this purpose. Moreover, the native genetic tools we developed provided valuable insights for further genetic modification of this probiotic yeast. Additionally, the *S. boulardii* strains we engineered displayed stable characteristics, and their antiviral activity was validated using a cell model, the results of which proved promising and are indicative of potential for further study in vivo. Overall, this research provides novel insights into the realm of developing probiotics into biotherapeutics.

## Figures and Tables

**Table 1:** Plasmids and strains.

Plasmids and Strains	Name	Descriptions	Source
Vector plasmid	pSF-TEF1-URA3	Shuttle vector plasmid of *E. coli* and yeast containing constitutive TEF1 promoter from *Saccharomyces cerevisiae* and *URA3* gene for auxotrophic selection.	Prof. Per E. J. Saris University of Helsinki, Helsinki, Finland
Recombinant plasmids for griffithsin secretion	pSF-TEF1-SED1-GRFT	Griffithsin expression plasmid containing TEF1 promoter and SED1 signal sequence from *S. boulardii*.	Designed in this study, constructed by GenScript Biotechnology Co., Ltd., Nanjing, China
	pSF-TEF1-αMF-GRFT	Griffithsin expression plasmid containing TEF1 promoter and αMF signal sequence from *S. boulardii*.	
	pSF-TEF1-STA1-GRFT	Griffithsin expression plasmid containing TEF1 promoter from *S. boulardii* and STA1 signal sequence from *S. diastaticus*.	
	pSF-TEF1-CL-GRFT	Griffithsin expression plasmid containing TEF1 promoter from *S. boulardii* and CL signal sequence from chicken lysozyme.	
	pSF-TDH3-SED1-GRFT	Griffithsin expression plasmid containing TDH3 promoter and SED1 signal sequence from *S. boulardii*.	
	pSF-TDH3-αMF-GRFT	Griffithsin expression plasmid containing TDH3 promoter and αMF signal sequence from *S. boulardii*.	
	pSF-TDH3-STA1-GRFT	Griffithsin expression plasmid containing TDH3 promoter from *S. boulardii* and STA1 signal sequence from *S. diastaticus*.	
	pSF-TDH3-CL-GRFT	Griffithsin expression plasmid containing TDH3 promoter from *S. boulardii* and CL signal sequence from chicken lysozyme.	
	pSF-PGK1-SED1-GRFT	Griffithsin expression plasmid containing PGK1 promoter and SED1 signal sequence from *S. boulardii*.	
	pSF-PGK1-αMF-GRFT	Griffithsin expression plasmid containing PGK1 promoter and αMF signal sequence from *S. boulardii*.	
	pSF-PGK1-STA1-GRFT	Griffithsin expression plasmid containing PGK1 promoter from *S. boulardii* and STA1 signal sequence from *S. diastaticus*.	
	pSF-PGK1-CL-GRFT	Griffithsin expression plasmid containing PGK1 promoter from *S. boulardii* and CL signal sequence from chicken lysozyme.	
Host strains	*S. boulardii* SAA940 (SAA940)	URA3-deficient strain of *Saccharomyces boulardii* CNCM I-745, host strain for griffithsin secretion	Prof. Vahid Khalaj, the Pasteur Institute of Iran, Tehran, Iran
	*E. coli* DH5α	Intermediate host strain for recombinant plasmids	Novizan Biotechnology Co., Ltd., Nanjing, China
RERecombinant *S. boulardii* strains	*S. boulardii* OG534 (OG534)	*S. boulardii* SAA940 carrying pSF-TEF1-URA3 (OG534), vector control strain	This study
	*S. boulardii* FE (FE)	*S. boulardii* SAA940 carrying plasmid pSF-TEF1-SED1-GRFT	
	*S. boulardii* FM (FM)	*S. boulardii* SAA940 carrying plasmid pSF-TEF1-αMF -GRFT	
	*S. boulardii* FT (FT)	*S. boulardii* SAA940 carrying plasmid pSF-TEF1-STA1-GRFT	
	*S. boulardii* FC (FC)	*S. boulardii* SAA940 carrying plasmid pSF-TEF1-CL-GRFT	
	*S. boulardii* HE (HE)	*S. boulardii* SAA940 carrying plasmid pSF-TDH3-SED1-GRFT	
	*S. boulardii* HM (HM)	*S. boulardii* SAA940 carrying plasmid pSF-TDH3-αMF-GRFT	
	*S. boulardii* HT (HT)	*S. boulardii* SAA940 carrying plasmid pSF-TDH3-STA1-GRFT	
	*S. boulardii* HC (HC)	*S. boulardii* SAA940 carrying plasmid pSF-TDH3-CL-GRFT	
	*S. boulardii* KE (KE)	*S. boulardii* SAA940 carrying plasmid pSF-PGK1-SED1-GRFT	
	*S. boulardii* KM (KM)	*S. boulardii* SAA940 carrying plasmid pSF-PGK1-αMF-GRFT	
	*S. boulardii* KT (KT)	*S. boulardii* SAA940 carrying plasmid pSF-PGK1-STA1-GRFT	
	*S. boulardii* KC (KC)	*S. boulardii* SAA940 carrying plasmid pSF-PGK1-CL-GRFT	

**Table 2:** Survival rates of selected yeast strains in the stomach, small intestine, and large intestine.

		*S. boulardii* Strains					
Digestive Fluids	Incubation Time	SAA940	OG534	FM	FT	HE	HC
Stomach	1 h	96.46 ± 1.62 ^ab^	97.46 ± 0.00 ^a^	91.85 ± 0.71 ^bc^	89.99 ± 2.24 ^c^	97.29 ± 0.80 ^a^	93.71 ± 3.52 ^abc^
	2 h	96.56 ± 1.43 ^a^	95.88 ± 0.00 ^a^	93.06 ± 2.26 ^ab^	86.32 ± 1.49 ^c^	96.51 ± 0.89 ^a^	91.25 ± 2.40 ^b^
Small intestine	2 h	96.72 ± 0.39 ^a^	97.27 ± 1.45 ^a^	89.18 ± 0.18 ^b^	89.21 ± 0.64 ^b^	97.49 ± 1.47 ^a^	89.05 ± 1.65 ^b^
	4 h	95.94 ± 0.68 ^a^	97.81 ± 1.31 ^a^	89.61 ± 0.43 ^b^	90.87 ± 1.71 ^b^	97.18 ± 0.61 ^a^	91.20 ± 1.65 ^b^
Large intestine	2 h	98.43 ± 1.65 ^a^	98.31 ± 1.00 ^a^	95.36 ± 1.94 ^a^	89.57 ± 1.14 ^b^	96.98 ± 0.66 ^a^	90.2 ± 2.64 ^b^
	4 h	97.47 ± 1.17 ^a^	98.74 ± 1.97 ^a^	93.79 ± 1.86 ^b^	89.4 ± 0.90 ^c^	97.41 ± 0.62 ^a^	90.8 ± 0.83 ^bc^
	6 h	97.68 ± 1.67 ^a^	97.03 ± 1.56 ^a^	91.64 ± 5.1 ^ab^	92.43 ± 1.37 ^ab^	97.18 ± 0.95 ^a^	90.35 ± 1.48 ^b^
	8 h	96.34 ± 0.79 ^a^	96.06 ± 0.00 ^a^	91.91 ± 4.38 ^ab^	92.32 ± 0.00 ^ab^	96.75 ± 0.00 ^a^	90.6 ± 1.62 ^b^

Note: Yeast strains were separately incubated in three different simulated digestive fluids, serially diluted, and plated onto agar media. Obtained colonies were counted (CFU) to determine the strains’ tolerance to gastrointestinal pressure. Survival rate% = (post-digestion CFU/initial CFU) × 100%. Data are presented as mean values with standard deviation. Duncan’s method was used for multiple comparisons of data between different groups; different letters in the same row indicate significant differences between different groups (*p* < 0.05).

## Data Availability

The original contributions presented in the study are included in the article/Supplementary Materials, further inquiries can be directed to the corresponding authors.

## Supplementary Materials

The following supporting information can be downloaded at https://www.mdpi.com/article/10.3390/microorganisms12122414/s1. Figure S1: Methylene blue staining of selected yeast strains; Table S1: Information of the signal sequences; Table S2: Information of the promoters.
