# Supplementary material for: Heterologous Expression of the Antiviral Lectin Griffithsin in Probiotic Saccharomyces boulardii and In Vitro Characterization of Its Properties
Source: Microorganisms. 2024 Nov 25;12(12):2414. doi: 10.3390/microorganisms12122414 (PMC11678560; doi:10.3390/microorganisms12122414)
Supplement: Supplementary file 1 [file microorganisms-12-02414-s001.zip › microorganisms-3256918-supplementary.pdf]

Supplementary Materials

Heterologous Expression of the Antiviral Lectin Griffithsin in Probiotic *Saccharomyces boulardii* and In Vitro Characterization of Its Properties

Jie Tang [1,†], Ran Li [2,*,†], Tingyu Jiang [2], Jiachen Lv [2], Yuwei Jiang [2], Xingjian Zhou [2], Hong Chen [2], Meiliang Li [2], Aimin Wu [1], Bing Yu [1], Timo M. Takala [3], Per E. J. Saris [3], Shuhong Li [2] and Zhengfeng Fang [1,2,*]

[1] Key Laboratory for Animal Disease-Resistance Nutrition of China Ministry of Education, Animal Nutrition Institute, Sichuan Agricultural University, Chengdu 611130, China; tangjie0422@foxmail.com (J.T.); wuaimin0608@163.com (A.W.); ybingtian@163.com (B.Y.)

[2] Key Laboratory of Agricultural Product Processing and Nutrition Health (Co-Construction by Ministry of Agriculture and Rural Affairs of China and Sichuan Province), College of Food Science, Sichuan Agricultural University, Ya'an 625014, China; tyjiang2003@163.com (T.J.); lvjiachen0321@foxmail.com (J.L.); jiapaxriver@foxmail.com (Y.J.); xingjianzhou210@foxmail.com (X.Z.); chenhong945@sicau.edu.cn (H.C.); liml@sicau.edu.cn (M.L.); lish@sicau.edu.cn (S.L.)

[3] Department of Microbiology, Faculty of Agriculture and Forestry, University of Helsinki, 00014 Helsinki, Finland; timo.takala@helsinki.fi (T.M.T.); per.saris@helsinki.fi (P.E.J.S.)

* Correspondence: liran@sicau.edu.cn (R.L.); zfang@sicau.edu.cn (Z.F.)

† These authors contributed equally to this work.

Supplementary Materials

Figures

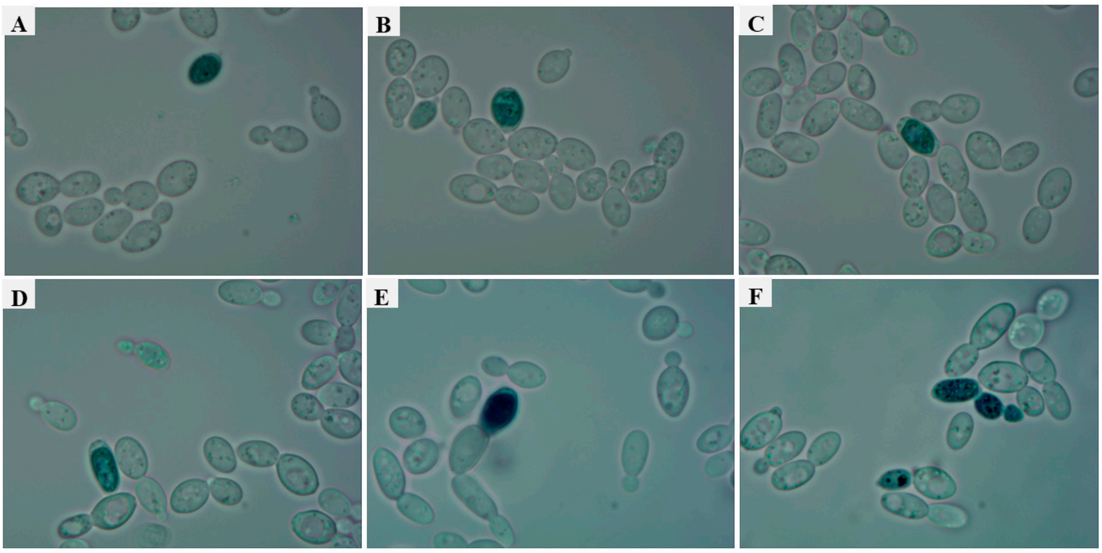

Figure S1. Methylene blue staining of selected yeast strains. A: *S. boulardii* SAA940, B: *S. boulardii* OG544, C: *S. boulardii* FM, D: *S. boulardii* FT, E: *S. boulardii* HE, F: *S. boulardii* HC.

Supplementary Materials

Tables

Table S1. Information of the signal sequences

**Signal sequences**	**Source**	**Size**	**DNA sequence**	**DNA sequence after codon optimization**	**Amino acid sequence**
SED1	630940-630996 bp in Chromosome IV (CM003540.1) of *S. boulardii* CNCM I-745	57 bp	ATGAAATTATCAACTGTCCTATTATCTGCCGG TTTAGCCTGGACTACTTTGGCCCAA	No optimization required	MKLSTVLLA GLAWTTLQ
αMF	33929-34195 bp in Chromosome XVI (CM003552.1) of *S. boulardii* CNCM I-745	267 bp	ATGAGATTTCCTTCAATTTTTACT GCAGTTTTATTCGCAGCATCCTCCGCATTAG CTGCTCCAGTCAACACTACAACAGAAGATG AAACGGCACAAATTCCGGCTGAAGCTGTCA TCGGTTACTTAGATTTAGAAGGGGATTTCGA TGTTGCTGTTTTGCCATTTTCCAACAGCACA AATAACGGGTTATTGTTTATAAATACTACTAT TGCCAGCATTGCTGCTAAAGAAGAAGGGGT ATCTTTGGATAAAAGAGAGGCTGAAGCT	No optimization required	MRFPSIFTAVLF AASSALAAPVN TTTEDETAQIPA EAVIGYLDLEGD FDVAVLPFSNST NNGLLFINTTIA SIAAKEEGVSLD KREAEA

Supplementary Materials

STA1	*Saccharomyces diastaticus* [56]	90 bp	ATGGTAGGCCTCAAAAATCCATATACGCACA CTATGCAAAGACCATTTCTACTCGCTTATTTG GTCCTTTCGCTTCTATTTAACTCAGCT	ATGGTTGGTTTGAAG AACCCATACACTCAC ACCATGCAAAGACCA TTCTTGTTGGCTTACT TGGTCTTGTCCCTATT ATTCAACTCTGCC	MVGLKNPYTHT MQRPFLLAYLV LSLLFNSA
CL	Chicken lysozyme signal peptide [49,57]	54 bp	ATGAGGTCTTTGCTAATCTTGGTGCTTTGCT TCCTGCCCCTGGCTGCTCTGGGG	ATGAGATCTCTATTAA TCTTGGTTTTGTGTTT CTTGCCATTGGCCGCT TTGGGT	MRSLLILVLCFL PLAALG

Supplementary Materials

Table S2. Information of the promoters

Promoters	Source	Reference	DNA sequence	Size
TDH3	198933-199576 bp in chromosome VII (CM003543.1) of *S. boulardii* CNCM I-745	GAP promoter from p426GPD plasmid	TCATTATCAATACTGCCATTTCAAAGAATACGTAAATAATTAATAGTAGTG ATTTTCCTAACTTTATTTAGTCAAAAAATTGGCCTTTTAATTCTGCTGTAAC CCGTACATGCCCAAAATAGGGGGCGGGTTACACAGAATATATAACATCAT AGGTGTCTGGGTGAACAGTTTATTCCTGGCATCCACTAAATATAATGGAG CCCGCTTTTTAAGCTGGCATCCAGAAAAAAAAAGAATCCCAGCACCAAA ATATTGTTTTCTTCACCAACCATCAGTTCATAGGTCCATTCTCTTAGCGCA ACTACACAGAACAGGGGCACAAACAGGCAAAAAACGGGCACAACCTCA ATGGAGTGATGCAACCTGCTTGGAGTAAATGATGACACAAGGCAATTGA CCTACGCATGTATCTATCTCATTTTCTTACACCTTCTATTACCTTCTGCTCTC TCTGATTTGGAAAAAGCTGAAAAAAAAAGGTTGAAACCAGTTCCCTGAAA TTATTCCCCTATTTGACTAATAAGTATATAAAGACGGTAGGTATTGATTGTA ATTCTGTAAATCTATTTCTTAAACTTCTTAAATTCTACTTTTATAGTTAGTCT TTTTTTTAGTTTTAAAACACTAAGAACTTAGTTTCGA	644 bp

Supplementary Materials

PGK1	135935-136891 bp in chromosome III (CM003539.1) of *S. boulardii* CNCM I-745	*S.cerevisiae* s288c genome (GenBank accession number: CP020125.1， nucleotides 142078－143034)	TTATCTTGTTTTGCAAGTACCACTGAGCAGGATAATAATAGAAATGATAAT<br>ATACTATAGTAGAGATAACGTCGATGACTTCCCATACTGTAATTGCTTTTA<br>GTTGTGTATTTTTAGTGTGCAAGTTTCTGTAAATCGATTAATTTTTTTTTCT<br>TTCCTCTTTTTATTAACCTTAATTTTTATTTTAGATTCCTGACTTCAACTCA<br>AGACGCACAGATATTATAACATCTGCATAATAGGCATTTGCAAGAATTACT<br>CGTGAGTAAGGAAAGAGTGAGGAACTATCGCATACCTGCATTTAAAGAT<br>GCCGATTTGGGCGCGAATCCTTTATTTTGGCTTCACCCTCATACTATTATCA<br>GGGCCAGAAAAAGGAAGTGTTTCCCTCCTTCTTGAATTGATGTTACCCTC<br>ATAAAGCACGTGGCCTCTTATCGAGAAAGAAATTACCGTCGCTCGTGATT<br>TGTTTGCAAAAAGAACAAAACTGAAAAAACCCAGACACGCTCGACTTC<br>CTGTCTTCCTATTGATTGCAGCTTCCAATTTCGTCACACAACAAGGTCCTA<br>GCGACGGCTCACAGGTTTTGTAACAAGCAATCGAAGGTTCTGGAATGGC<br>GGGAAAGGGTTTAGTACCACATGCTATGATGCCCACTGTGATCTCCAGAG<br>CAAAGTTCGTTCGATCGTACTGTTACTCTCTCTCTTTCAAACAGAATTGTC<br>CGAATCGTGTGACAACAACAGCCTGTTCTCACACACTCTTTTCTTCTAAC<br>CAAGGGGGTGGTTTAGTTTAGTAGAACCTCGTGAAACTTACATTTACATA<br>TATATAAACTTGCATAAATTGGTCAATGCAAGAAATACATATTTGGTCTTTT	957 bp

Supplementary Materials

			CTAATTCTTAGTTTTTCAAGTTCTTAGATGCTTTCTTTTTCTCTTTTTTACA<br>GATCATCAAGGAAGTAATTATCTACTTTTTACAACAAATATAAAAC	
TEF1	539901-540478 bp in chromosome XVI (CM003552.1) of *S. boulardii* CNCM I-745	pSF-OG534-URA3	CAATGCATACTTTGTACGTTCAAAATACAATGCAGTAGATATATTTATGCAT<br>ATTACATATAATACATATCACATAGGAAGCAACAGGCGCGTTGGACTTTTA<br>ATTTTCGAGGACCGCGAATCCTTACATCACACCCAATCCCCCACAAGTGA<br>TCCCCCACACACCATAGCTTCAAAATGTTTCTACTCCTTTTTACTCTTCC<br>AGATTTTCTCGGACTCCGCGCATCGCCGTACCACTTCAAAACACCCAAGC<br>ACAGCATACTAAATTTCCCCTCTTTCTTCCTCTAGGGTGTCGTTAATTACC<br>CGTACTAAAGGTTTGGAAAAGAAAAAAGAGACCGCCTCGTTTCTTTTTC<br>TTCGTCGAAAAAGGCAATAAAAATTTTATCACGTTTCTTTTTCTTGAAA<br>ATTTTTTTTTTTGATTTTTTTCTCTTTCGATGACCTCCCATTGATATTTAAGT<br>TAATAAACGGTCTTCAATTTCTCAAGTTTCAGTTTCATTTTTCTTGTTCTAT<br>TACAACTTTTTTTACTTCTTGCTCATTAGAAAGAAAGCATAGCAATCTAAT<br>CTAAGTTTTAATTACAAA	578 bp
